# Dissecting the Role of Serotonin in Drought Responses of Rocket Plants: From Serotonin Accumulation to Tryptophan–ABA Associations

**DOI:** 10.1111/ppl.71106

**Published:** 2026-09-04

**Authors:** Sofía Álvarez‐Real, Sergi Munné‐Bosch, Alba Arabia

**Affiliations:** ^1^ Department of Evolutionary Biology, Ecology and Environmental Sciences University of Barcelona Barcelona Spain; ^2^ Nutrition and Food Safety Research Institute (INSA), University of Barcelona Barcelona Spain

**Keywords:** abscisic acid, drought stress, *Eruca vesicaria*, indoleamines, tryptophan

## Abstract

In recent years, serotonin has been proposed as a signaling molecule in several plant physiological processes, although evidence supporting this function remains limited to date. In particular, its involvement under water deficit conditions is poorly characterised, especially regarding its mechanism of action. This study aimed to determine the implication of serotonin in the response to water stress in rocket plants through three experimental approaches: (1) analysis of endogenous concentrations of indoleamines, tryptophan, serotonin, and melatonin during the acclimation response to stress, (2) evaluation of the dose‐dependent effect following its application in seedlings, and (3) analysis of the impact of serotonin application under field conditions. The results showed an increase in serotonin content under water deficit, which was negatively correlated with plant biomass and stomatal conductance, and positively correlated with abscisic acid (ABA) levels. Serotonin application in seedlings led to an improvement in plant water status in a dose‐dependent manner but did not alter ABA content. By contrast, this positive effect was not observed under crop conditions. Moreover, when all datasets were integrated, tryptophan, the precursor of serotonin, exhibited a stronger correlation with ABA than serotonin. Taken together, these findings indicate that serotonin does not appear to play a central role in regulating ABA‐mediated drought responses. Instead, the stronger association of tryptophan with ABA, together with its role as a precursor of indolamines and auxin, supports a more prominent role for tryptophan metabolism in the drought response than for serotonin itself.

## 1 Introduction

Plants are increasingly exposed to a multitude of abiotic stresses, including drought, salinity, and extreme temperatures, whose frequency and intensity are rising amid ongoing global climate change (Zandalinas et al. 2021). Among these, water deficit is one of the most prevalent and recurrent abiotic stresses in agricultural regions worldwide, posing a significant threat to agricultural sustainability and food security (Tramblay et al. 2020). Water scarcity negatively affects plant growth and development, leading to reduced biomass accumulation via stomatal closure and decreased photosynthetic activity (Mathobo et al. 2017; Hao et al. 2019). Plant responses to water deficit involve complex physiological, biochemical, and molecular processes that determine survival, growth, and ultimately agricultural productivity. Consequently, plants have evolved sophisticated regulatory networks to cope with such conditions, making it essential to understand the roles of key biomolecules that modulate these physiological responses (Chaudhry and Sidhu 2022). Accumulating evidence demonstrates that phytohormones alleviate stress‐induced damage through signal transduction pathways activating defence responses (Ullah et al. 2018; Jogawat et al. 2021; Liao et al. 2025). Among phytohormones, abscisic acid (ABA) is the main stress‐related hormone, which induces the major adaptive mechanisms under water stress conditions by regulating transpiration efficiency, hydraulic conductivity and leaf growth (Thompson et al. 2007; Zandalinas et al. 2016; Wei et al. 2024). Moreover, stress responses are mediated not only by ABA but also by a complex phytohormonal crosstalk network. This includes interactions between ABA and other stress‐related hormones such as jasmonic acid (JA) and salicylic acid (SA) (Brossa et al. 2011; Muñoz‐Espinoza et al. 2015), as well as its crosstalk with growth‐related hormones, such as auxin, a tryptophan‐derived indoleamine contributing to the bidirectional regulation between growth and stress responses (Sun et al. 2018; Xu et al. 2013; Xiong et al. 2025).

Among indoleamines, serotonin and melatonin, both well‐known as neurotransmitters in vertebrates, have received growing attention in studies addressing plant stress responses, particularly in the case of melatonin (Arnao and Hernández‐Ruiz 2015; Erland et al. 2015). Over the last few decades, their potential role in plants has been increasingly investigated, suggesting roles as signaling molecules, and in the case of melatonin, even as a novel phytohormone (Mishra and Sarkar 2023; Abbasi et al. 2020; Chen and Arnao 2022; Arnao and Hernández‐Ruiz 2019). In plants, its biosynthetic pathway has been well‐characterised, deriving from tryptophan, which is first converted into tryptamine by tryptophan decarboxylase and is subsequently hydroxylated by tryptamine‐5‐hydroxylase to form serotonin. Serotonin acts as a central intermediate in the pathway leading to melatonin through sequential reactions catalysed by serotonin N‐acetyltransferase, N‐acetylserotonin O‐methyltransferase, and caffeic acid O‐methyltransferase (Back et al. 2016). Given that tryptophan serves as a precursor for multiple bioactive metabolites, its metabolism has emerged as a key regulatory hub in plant stress responses (Wang et al. 2025). Considerable progress has been made in understanding melatonin's role in stress responses, with numerous studies showing that it enhances stress tolerance through the enhancement of antioxidant systems, preservation of photosynthetic function, and modulation of hormonal signaling pathways (Noor and Sheng 2026; Harimadhav et al. 2026; Khan et al. 2024; Tiwari et al. 2021; Arnao and Hernández‐Ruiz 2018).

In contrast, although serotonin was first reported in plants prior to melatonin (Bowden et al. 1954) and is sometimes found at higher concentrations (Negri et al. 2021), it has received considerably less attention, likely due to its role as an intermediate in the melatonin biosynthetic pathway. Nevertheless, experimental evidence suggests that serotonin may be involved in various physiological processes, especially the regulation of root growth and architecture (Pelagio‐Flores et al. 2011; Wan et al. 2018), through the modulation of ROS accumulation and phytohormone biosynthesis and signaling pathways, such as auxins, JAs, and ethylene (Pelagio‐Flores et al. 2011, 2016). In addition, most evidence regarding its role in abiotic stress responses has emerged within the last 5 years. Exogenous serotonin has been shown to enhance antioxidant enzymes such as superoxide dismutase, catalase, and glutathione reductase, thereby decreasing malondialdehyde content and improving tolerance to salt and drought stress in rapeseed and tomato, respectively (Liu et al. 2021; Akcay and Okudan 2023). Indeed, serotonin has also been reported to alleviate growth inhibition by promoting mesocotyl and coleoptile elongation in maize (Zhao et al. 2023), increasing proline and soluble sugar accumulation under cold and salt stress in rapeseed (He et al. 2021; Liu et al. 2021), and elevating chlorophyll content in mustard (Zahra et al. 2024). Nevertheless, little is known about the mechanism of action of serotonin during stress, including its crosstalk with phytohormones. Current knowledge is mainly based on studies of root development, where serotonin has an anti‐auxin activity (Pelagio‐Flores et al. 2011) and salt stress resistance, where Lu et al. (2022) demonstrated that serotonin acts downstream of ABA in regulating root suberisation in rice, negatively modulating this process in response to salinity and thereby reducing stress tolerance. Indeed, these findings contrast with other studies reporting a positive role of serotonin in enhancing stress tolerance. Therefore, the involvement of serotonin in stress responses beyond a metabolic intermediate remains largely unresolved, particularly under other relevant abiotic stresses such as water deficit and in terms of its mechanism of action.

In this context, due to the limited current knowledge on serotonin in drought stress responses, this study investigates its role under water deficit in rocket ( *Eruca vesicaria* subsp. *sativa*). Rocket is a native Mediterranean species of the Brassicaceae family, widely consumed worldwide due to its high nutritional value and distinctive, slightly peppery flavor (Cavaiuolo and Ferrante 2014). As a fast‐growing annual herb with high water and nutrient demands, rocket is particularly sensitive to water deficit, which can lead to irreversible negative effects on growth, thereby threatening sustainable crop production and resulting in significant yield losses. Accordingly, we determined the role of serotonin under severe water deficit through three approaches: (1) analysis of endogenous indoleamines, including tryptophan, serotonin, and melatonin, during drought stress in rocket seedlings; (2) evaluation of the responses of seedlings to different doses of exogenous serotonin, focusing on the physiological and biochemical mechanisms underlying stress tolerance, including its impact on phytohormone content; and (3) assessment of the effects of serotonin application under field conditions to evaluate its potential as a biostimulant for enhancing stress tolerance in crops.

## 2 Materials and Methods

### 2.1 Plant Material and Growth Conditions

Rocket ( *Eruca vesicaria* subsp. *sativa*) seeds were provided by Semillas Fitó (Barcelona, Spain) and sown in a growth chamber (IBERCEX ASL walk‐in) under a 16/8 h (light/dark) photoperiod at 125 μmol m^−2^ s^−1^, 22°C, and 70% relative humidity at the Experimental Field Facilities of the University of Barcelona (Barcelona, NE Spain). At the three‐leaf stage, healthy and uniform seedlings were transplanted into individual 280 cm^3^ pots containing peat:perlite:vermiculite (2:1:1) and, after 3 days of acclimation, transferred to a semi‐controlled greenhouse.

### 2.2 Experimental Design, Treatments and Sampling

#### 2.2.1 First Experimental Approach: Rocket Seedlings Exposed to Water Stress

Following transfer to the greenhouse, all plants were irrigated for 1 week with 50% Hoagland nutrient solution (Hoagland and Arnon 1950). Thereafter, plants were divided into two irrigation regimes: well‐watered, which continued to receive irrigation, and water‐stressed, in which irrigation was completely withheld to induce water deficit. Samples were collected at 7, 10, and 14 days after the onset of the stress treatment. At each sampling point, 10 plants per treatment (*n* = 10) were randomly selected, and a range of stress‐related physiological markers (described below) were analysed. Moreover, leaf material was immediately frozen in liquid nitrogen and stored at −80°C for subsequent analysis of indolamines (tryptophan, serotonin, and melatonin) and phytohormone content.

#### 2.2.2 Second Experimental Approach: Dose‐Dependent Serotonin Application in Rocket Seedlings

For the second experimental approach, serotonin was applied as a foliar spray 7 days after the onset of water restriction in both well‐watered and water‐stressed plants at concentrations of 0, 1, 10, 50, and 100 μM (98%, Thermo Scientific; reference B21263), containing 0.1% (v/v) Tween 20 to enhance leaf absorption. Sampling was performed at 14 days after water restriction (7 days after serotonin application). At this sampling point, 10 seedlings per treatment (*n* = 10) were randomly selected according to serotonin concentration and irrigation regime to evaluate the effect of exogenous serotonin on physiological stress markers. In addition, leaf material was immediately frozen in liquid nitrogen and stored at −80°C for subsequent analysis of indolamines and phytohormone content.

#### 2.2.3 Third Experimental Approach: Exogenous Serotonin Application Under Field Conditions

Rocket seedlings were grown under semi‐controlled greenhouse conditions until the development of five true leaves. At this stage, plants were transferred to an experimental tunnel and grown in Calcic Luvisol soil under a UV‐resistant polyethylene cover. The tunnel was laterally open to allow air circulation while preventing direct rainfall. Two weeks after transfer, water deficit conditions were imposed in water‐stressed plants by withholding irrigation until harvest, whereas well‐watered plants were irrigated once a week for 10–30 min, depending on climatic conditions. One week after the onset of water deficit, serotonin treatments were initiated in both well‐watered and water‐stressed plants. Serotonin was applied at 100 μM (98%, Thermo Scientific; reference B21263) in 0.1% (v/v) Tween 20 solution to facilitate foliar uptake and sprayed onto the foliage. Treatments were applied twice, with a second application performed 1 week after the first using the same procedure. All applications were carried out in the late evening under dark conditions to minimise evaporation and photodegradation of the active compound. Sampling was performed at the commercial harvest stage, 1 week after the second application. At this time point, stress‐related physiological markers were evaluated, and leaf material was immediately frozen in liquid nitrogen and stored at −80°C for subsequent analysis of phytohormones and indolamines.

### 2.3 Stress‐Related Physiological Markers

In the three experimental approaches, stomatal conductance (gs; mmol m^−2^ s^−1^) was first measured in situ on fully developed leaves under field conditions using a LI‐COR 6400XT system (LI‐COR Biosciences). Subsequently, plants were harvested to determine shoot biomass (g). From the same leaves used for stomatal conductance measurements, the maximum quantum efficiency of photosystem II (Fv/Fm) was assessed after 1 h of dark acclimation using a Mini‐PAM II Photosynthesis Yield Analyzer (Walz), and relative water content was also calculated as 100 × (FW − DW)/(TW − DW), where FW is fresh weight, TW is turgid weight after 24 h of immersion in distilled water at 4°C, and DW is dry weight after oven‐drying at 80°C until constant weight.

Photosynthetic pigments, including chlorophyll a, chlorophyll b, and total carotenoids, were determined using a double‐beam UV/Visible spectrophotometer (CE7400 Aquarius, Cecil Instruments Ltd.). Methanolic extracts were prepared from 100 mg of well‐powdered ground frozen rocket leaf tissue and diluted 1:10 (v/v) or 1:20 (v/v) when required. Absorbance was measured at 470, 653, 666, and 750 nm, and total chlorophyll and carotenoid contents were calculated following the equations of Lichtenthaler and Wellburn (1983).

### 2.4 Analysis of Indolamines and Phytohormones Content

Serotonin, its precursor tryptophan, and its product melatonin, were extracted and quantified, together with stress‐ and growth‐related phytohormones, including abscisic acid (ABA), jasmonates [12‐*oxo*‐phytodienoic acid (OPDA), jasmonic acid (JA) and the conjugated form jasmonoyl isoleucine (JA‐Ile)], salicylic acid (SA), indole‐3‐acetic acid (IAA), cytokinins [2‐isopentenyl adenine (2iP), trans‐zeatin (t‐Z)], and gibberellin 4 (GA_4_), by ultrahigh‐performance liquid chromatography coupled to electrospray ionisation tandem mass spectrometry (UHPLC/ESI‐MS/MS). Ground and frozen rocket leaf (100 mg) was extracted with 400 μL cold 100% (v/v) methanol containing deuterium‐labeled indoleamines and hormones (Olchemim Ltd.) as internal standards. The extracts were subjected to ultrasonication (Branson 3800 ultrasonic cleaner, Bransonic) in an ice bath at 4°C for 30 min, followed by a 10 min centrifugation at 15980 *g* at 4°C (Labnet International Inc.). The supernatant was collected, and the remaining pellet was subjected to two successive re‐extractions with 300 μL of cold methanol each. All supernatants were combined and the final extract was filtered through 0.22 μm hydrophobic PTFE filters (Phenomenex). UHPLC was coupled to a triple quadrupole mass spectrometer (QTRAP 6500, AB Sciex, Concord) for analyses, as described in Arabia et al. (2025). Quantification was made considering recovery rates for each sample by using the deuterium‐labeled internal standards. For tryptophan and melatonin, d_4_‐melatonin (178 m/z, 21.8 V CE) was used, whereas d_4_‐serotonin (181.1 m/z, 17.0 V CE) was used for serotonin quantification. Finally, calibration curves for each analyte were generated using MultiQuantTM 3.0.1 software.

### 2.5 Statistical Analyses

For the first and second experimental approaches, a two‐way analysis of variance (ANOVA) was performed to determine the effects of ‘Irrigation (I)’, ‘Time (T)’ or ‘Serotonin Treatment (Tr)’ and the interaction between these factorial variables. For the third experimental approach, a three‐way ANOVA was performed to identify the effects of ‘Condition (C)’, ‘Irrigation (I)’, ‘Serotonin Treatment (Tr)’ and interactions. In all data, multiple comparisons were conducted when the interaction showed significant differences using the Duncan post hoc test. Data were transformed using log when normality and homoscedasticity were rejected, and if any transformation was effective, nonparametric ArtANOVA tests were performed. *p* < 0.05 was considered statistically significant. For all analysed parameters, Spearman correlations were conducted and considered significant at a probability level of *p < 0.05, **p < 0.01 and ***p < 0.001. All analyses were performed using RStudio. Graphs were created using SigmaPlot (System Software).

## 3 Results

### 3.1 Physiological Responses of Rocket Seedlings to Severe Water Deficit

**FIGURE 1:**
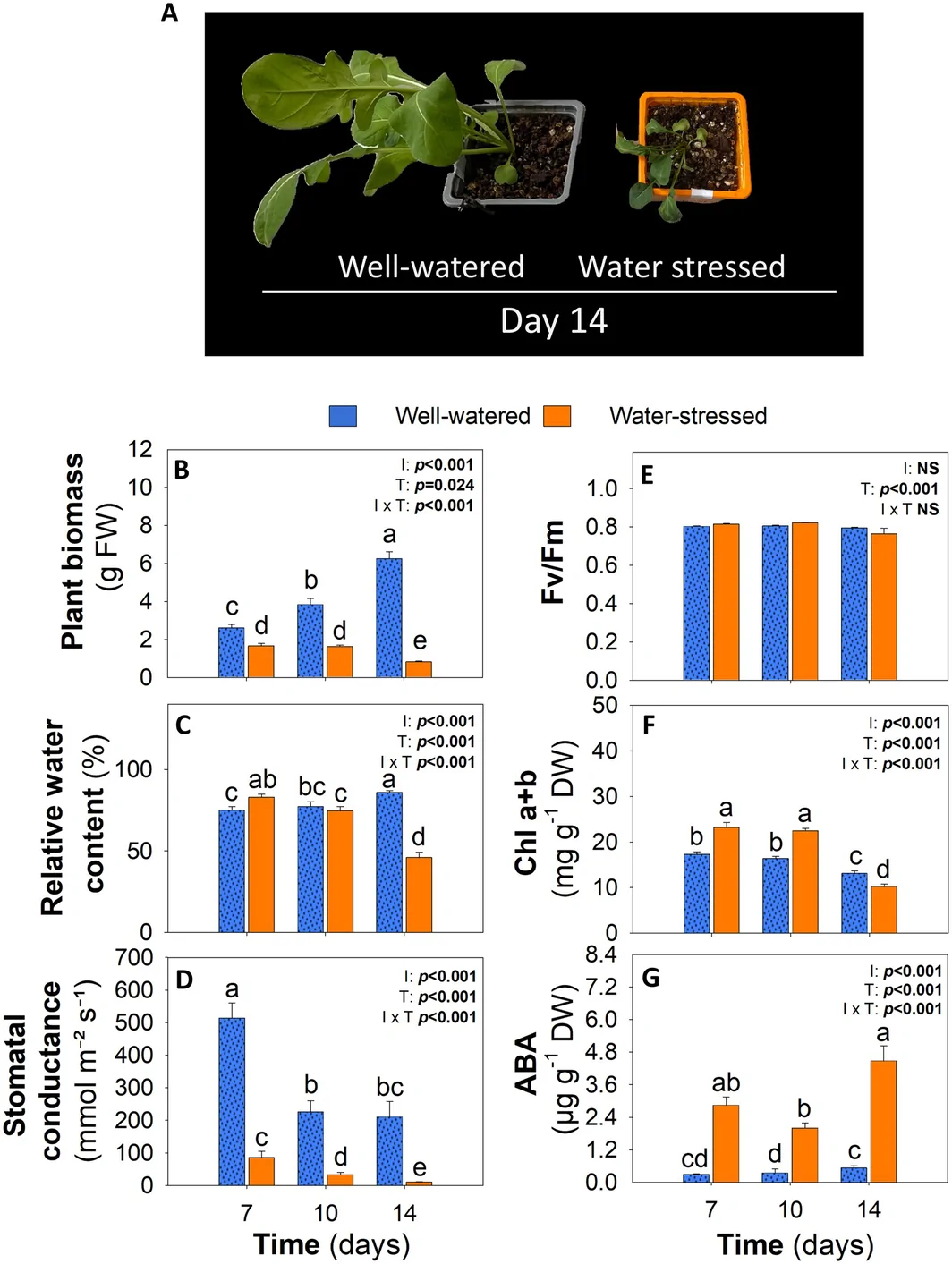
(A) Representative images of well‐watered and water‐stressed plants. (B) Plant biomass. (C) Relative water content. (D) Stomatal conductance. (E) Maximum efficiency of PSII (Fv/Fm). (F) Total chlorophyll content (Chl a + b). (G) Abscisic acid (ABA) content after 7, 10 and 14 days of water restriction in well‐watered and water‐stressed plants. Data are presented as mean ± SE (*n* = 10). Different lowercase letters indicate significant differences (*p* < 0.05, Duncan's post hoc test). I, irrigation; T, time; NS indicates no significant differences (*p* > 0.05).

Rocket plants exhibited a marked reduction in growth and physiological performance under severe drought conditions (Figure 1A). Water deficit drastically reduced shoot biomass by 85% after 14 days of water restriction compared to irrigated plants. However, a substantial early effect was already evident after 7 days of stress, with a 36% reduction in shoot biomass compared with well‐watered seedlings (Figure 1B). Moreover, this reduction in shoot biomass was accompanied by a decrease in relative water content, which declined by almost 50% at the point of maximum stress (14 days of water restriction) (Figure 1C).

Water deficit also induced a marked reduction in stomatal conductance throughout the stress period, decreasing by 85% after 7 and 10 days of treatment compared with irrigated seedlings, and becoming even more pronounced at the end of the experiment, reaching a 95% reduction after 14 days (Figure 1D). Furthermore, no significant changes in Fv/Fm were observed during the stress period, with values remaining above 0.75 throughout the experiment in both well‐watered and water‐stressed seedlings (Figure 1E). Total chlorophyll content showed a slight decrease at Day 14 (Figure 1F), which was accompanied by an increase in the carotenoid/chlorophyll ratio (Figure S1B). Indeed, carotenoid content was higher at days 7 and 10, although this increase was not maintained at Day 14 (Figure S1A). Furthermore, ABA, a key phytohormone involved in plant responses to water stress, showed a strong increase throughout the experiment under drought conditions, reaching 10‐, 6‐, and 8‐fold higher levels at 7, 10, and 14 days after the onset of stress, respectively (Figure 1G).

**FIGURE 2:**
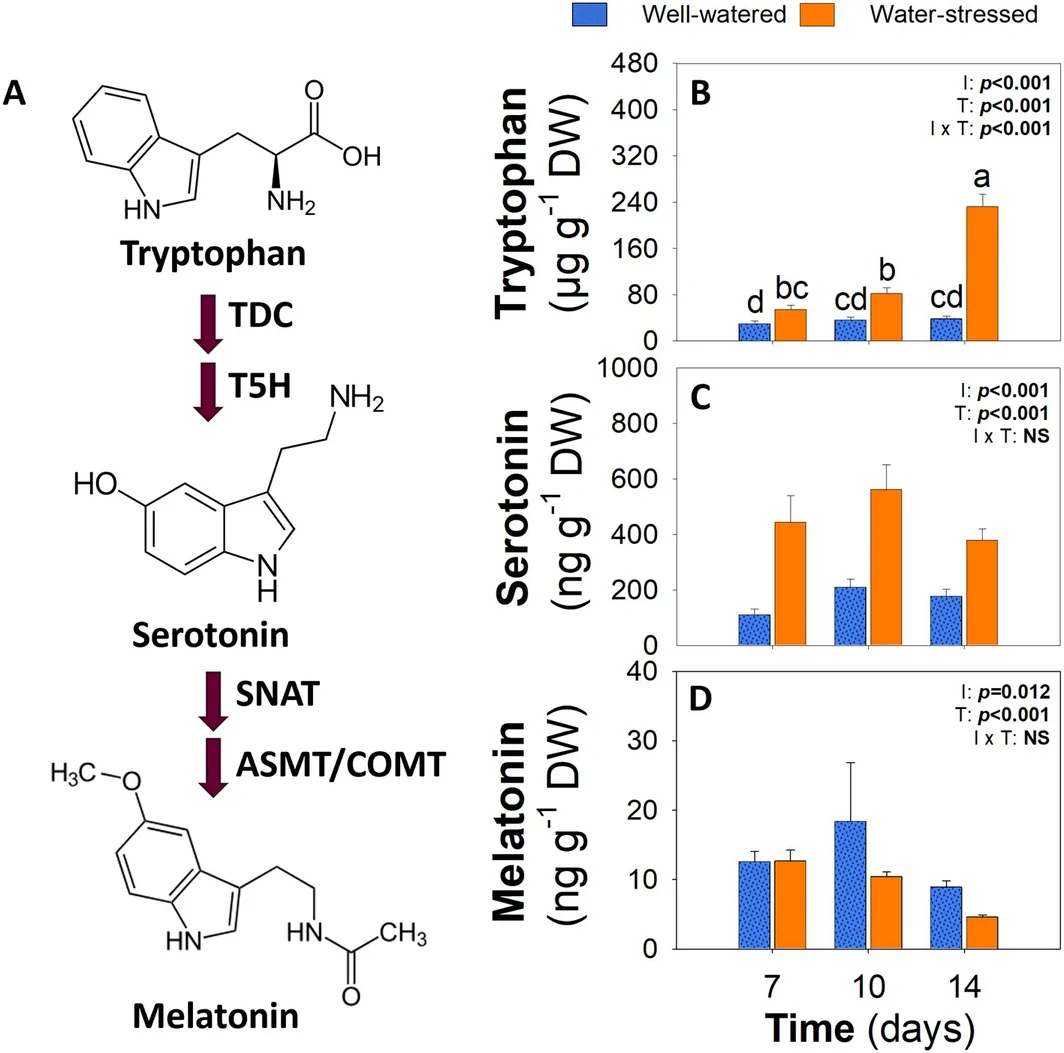
(A) Schematic representation of the indoleamine biosynthetic pathway. (B) Endogenous tryptophan content (C) Endogenous serotonin content (D) Endogenous melatonin content after 7, 10 and 14 days of water restriction in well‐watered and water‐stressed plants. Data are represented as mean ± SE (*n* = 10). Different lowercase letters indicate significant differences (*p* < 0.05, Duncan's post hoc test). I, irrigation; T, time; NS indicates no significant differences (*p* > 0.05). ASMT, acetylserotonin O‐methyltransferase; COMT, caffeic acid O‐methyltransferase; T5H, tryptamine 5‐hydroxylase; TDC, tryptophan decarboxylase; SNAT, serotonin N‐acetyltransferase.

Moreover, drought‐induced changes in the content of the analysed indolamines in rocket plants (Figure 2A). In irrigated plants, the concentrations of tryptophan, serotonin, and melatonin remained stable throughout the experiment. In contrast, stressed plants showed higher levels of tryptophan and serotonin compared with well‐watered plants (Figure 2B–D). Serotonin levels were consistently higher during the entire stress period, showing a 2‐fold increase relative to irrigated plants (Figure 2C). Likewise, during the stress period, endogenous tryptophan levels also remained elevated in water‐stressed seedlings, particularly after 14 days of water restriction, when a 6‐fold increase was observed compared with well‐watered plants (Figure 2B). In contrast, melatonin levels remained stable in rocket seedlings throughout the experiment (Figure 2D), and its absolute concentrations were consistently lower than those of serotonin, with irrigated plants showing values of approximately 10 ng g^−1^ DW for melatonin compared with around 100 ng g^−1^ DW for serotonin.

**FIGURE 3:**
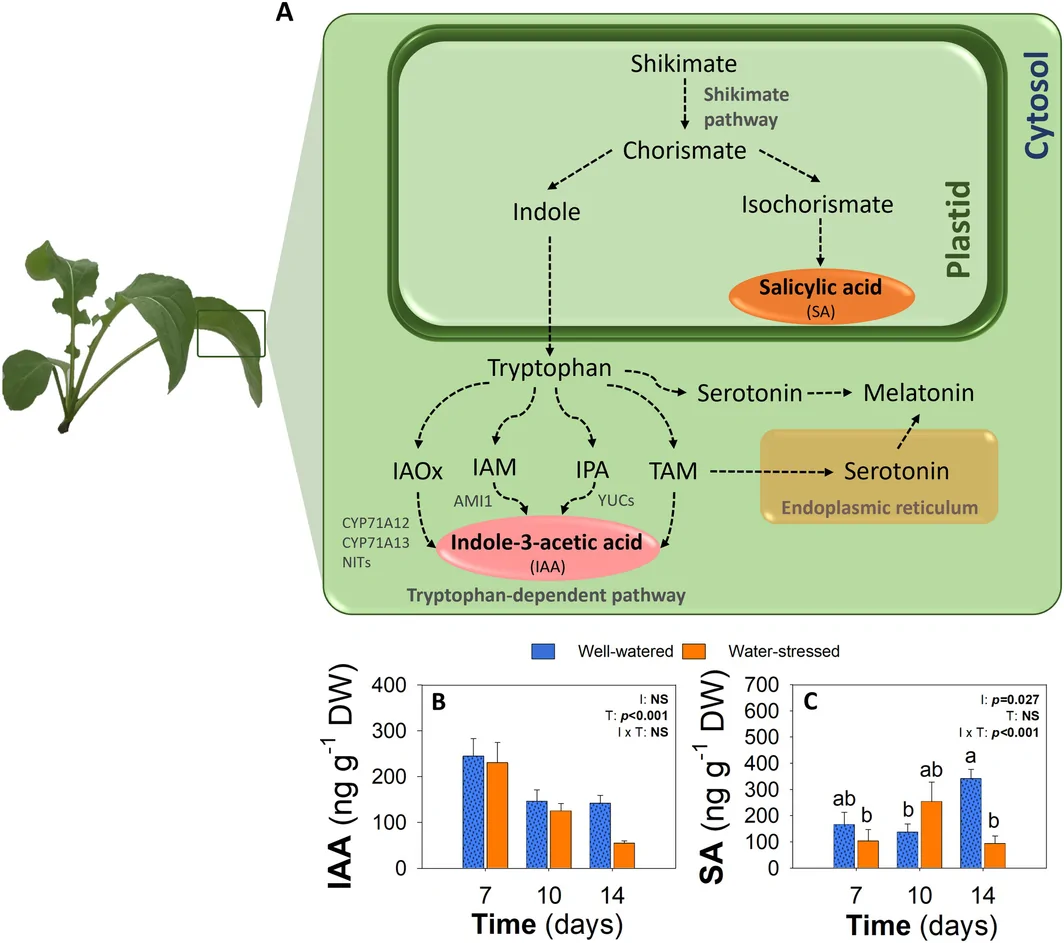
(A) Schematic representation of the indole‐3‐acetic acid (IAA) and salicylic acid (SA) biosynthetic pathways. (B) Endogenous IAA content. (C) Endogenous SA content after 7, 10, and 14 days of water restriction in well‐watered and water‐stressed plants. Data are represented as mean ± SE (*n* = 10). Different lowercase letters indicate significant differences (*p* < 0.05, Duncan's post hoc test). I, irrigation; T, time; NS indicates no significant differences (*p* > 0.05). IAM, indole‐3‐acetamide; IAOx, indole‐3‐acetaldoxime; IPA, indole‐3‐pyruvic acid; TAM, tryptamine.

The content of other indole‐related compounds derived from tryptophan, such as IAA, was also analysed, together with SA, which is derived from chorismate and therefore shares metabolic precursors with serotonin (Figure 3A). IAA levels were not affected by water stress and showed a general decrease over time in both well‐watered and water‐stressed plants (Figure 3B). Regarding SA, no clear temporal pattern was observed. However, at the point of maximum stress, water‐stressed plants showed a 70% reduction in SA levels (Figure 3C). No significant changes were observed in the other analysed hormones (Figure S1D–I).

A Spearman correlation matrix (*n* = 60) was constructed to assess relationships among physiological parameters, phytohormones, and tryptophan‐derived metabolites (Figure S2). Results showed that ABA was strongly and negatively correlated with stomatal conductance (*r* = −0.78, *p* < 0.001) and plant biomass (*r* = −0.72, *p* < 0.001). Serotonin also exhibited significant negative correlations with stomatal conductance (*r* = −0.66, *p* < 0.001), plant biomass (*r* = −0.50, *p* < 0.01), and a positive correlation with ABA (*r* = 0.61, *p* < 0.001). These relationships were even stronger for tryptophan, which showed a correlation with stomatal conductance similar to that observed for ABA (*r* = −0.78, *p* < 0.001), a significant negative correlation with plant biomass (*r* = −0.58, *p* < 0.01), and a stronger positive association with ABA levels (*r* = 0.71, *p* < 0.001) compared with serotonin.

Additionally, tryptophan and serotonin showed weaker but significant negative correlations with IAA, whereas melatonin was positively correlated with IAA (*r* = 0.64, *p* < 0.001) and all the other growth‐related phytohormones analysed (Figure S2).

### 3.2 Dose‐Dependent Effects of Serotonin Application Under Water Stress in Seedlings

**FIGURE 4:**
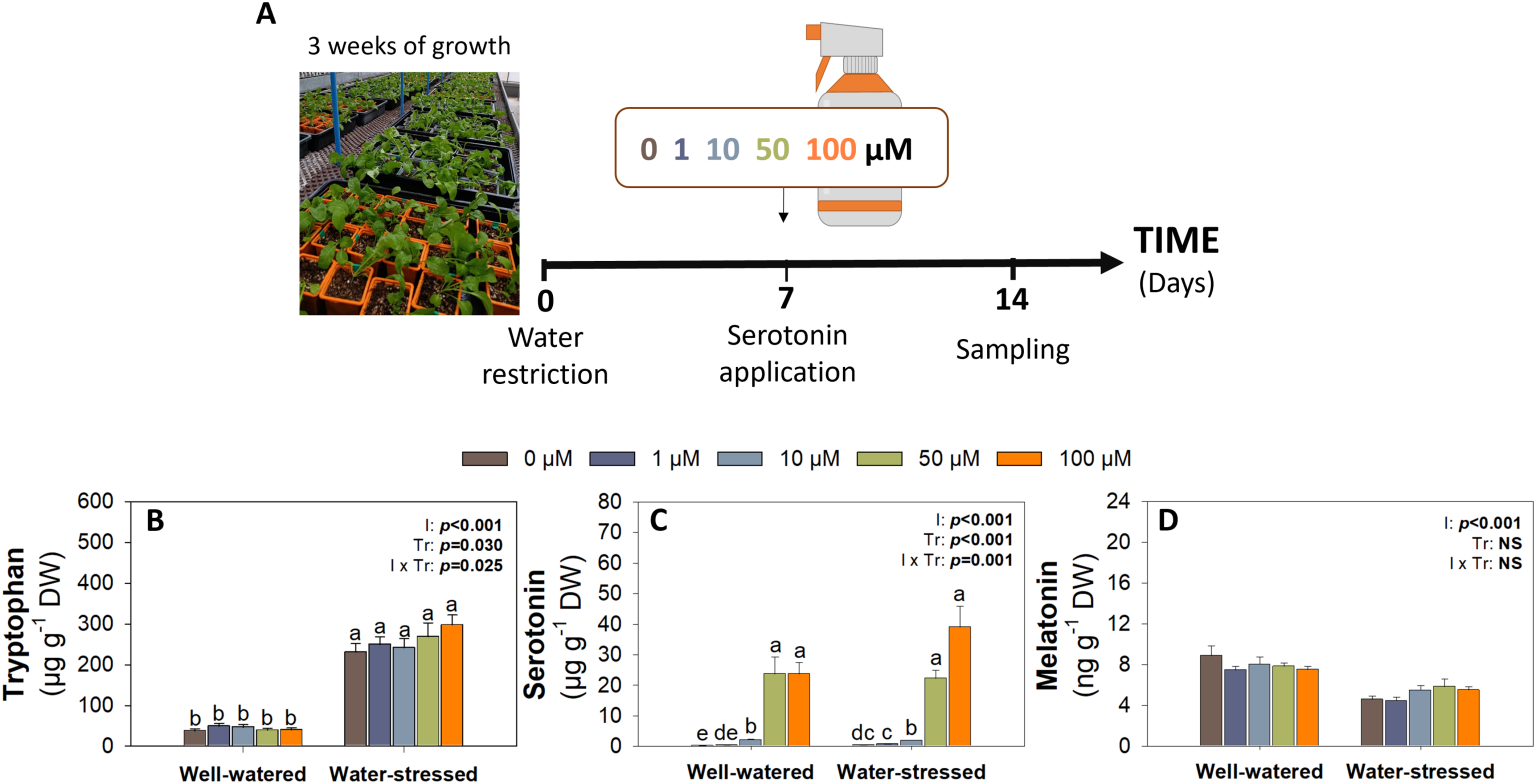
(A) Experimental design showing serotonin application (0, 1, 10, 50, and 100 μM) and the water restriction period. (B) Endogenous tryptophan content. (C) Endogenous serotonin content. (D) Endogenous melatonin content after 14 days of water restriction and 7 days after serotonin treatment in well‐watered and water‐stressed plants. Data are presented as mean ± SE (*n* = 10). Different lowercase letters indicate significant differences (*p* < 0.05, Duncan's post hoc test). I, irrigation; Tr, serotonin treatment; NS indicates no significant differences (*p* > 0.05).

Exogenous serotonin was applied at different concentrations (0, 1, 10, 50, and 100 μM) under both irrigation regimes and its effects on indoleamine pathway metabolites were analysed in rocket plants (Figure 4A). Serotonin content increased in a dose‐dependent manner in both well‐watered and water‐stressed plants, reaching up to a 100‐fold increase at the highest concentration (100 μM) compared with untreated controls (Figure 4C). In contrast, neither tryptophan nor melatonin levels were significantly affected by serotonin treatments, with no changes observed across concentrations (Figure 4B–D).

**FIGURE 5:**
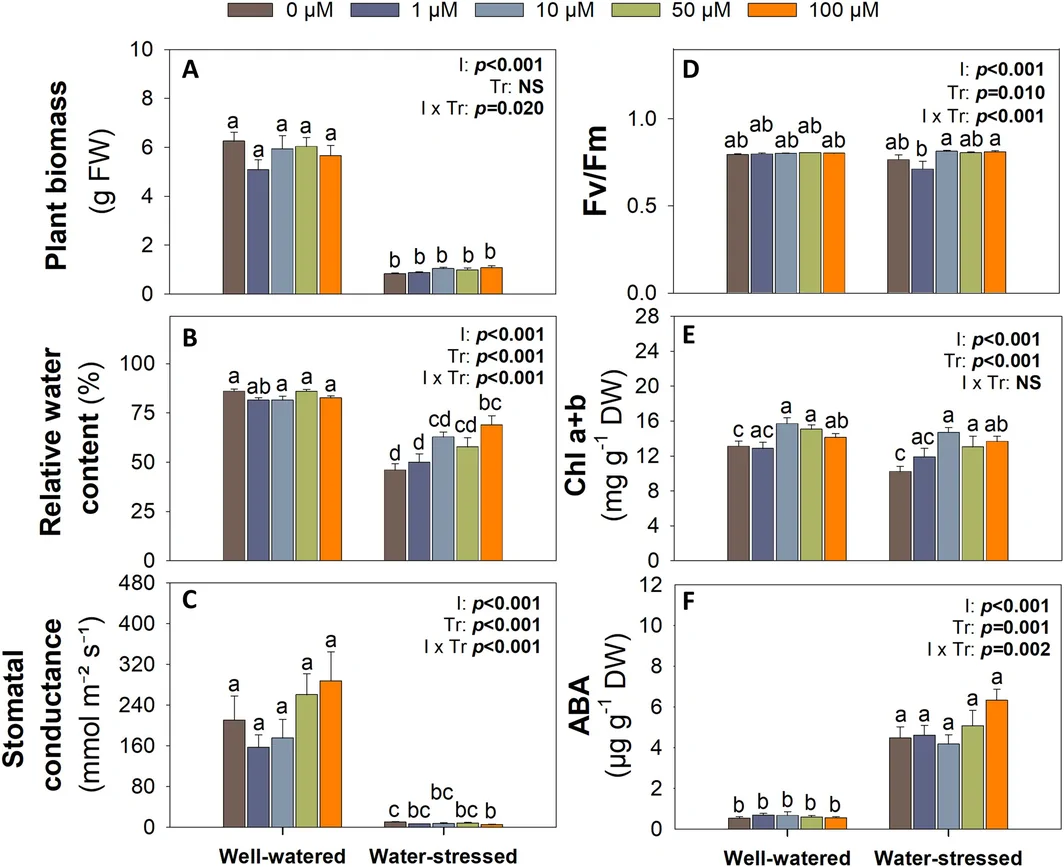
(A) Plant biomass. (B) Relative water content. (C) Stomatal conductance. (D) Maximum efficiency of PSII (Fv/Fm). (E) Total chlorophyll content (Chl a + b). (F) Abscisic acid (ABA) content after 14 days of water restriction and 7 days after serotonin treatment in well‐watered and water‐stressed plants. Data are presented as mean ± SE (*n* = 10). Different lowercase letters indicate significant differences (*p* < 0.05, Duncan's post hoc test). I, irrigation; Tr, serotonin treatment; NS indicates no significant differences (*p* > 0.05).

The analysis of physiological stress indicators after exogenous serotonin applications showed that serotonin improved relative water content in water‐stressed seedlings. This effect was dose‐dependent, with the highest improvement observed at 100 μM, where it increased by 33% compared with untreated water‐stressed plants (Figure 5B). In addition, stomatal conductance decreased by 48% in water‐stressed plants treated with 100 μM serotonin compared with untreated stressed plants, whereas no significant effects were observed at lower concentrations (Figure 5C). Similarly, exogenous serotonin increased total chlorophyll content independently of irrigation regime at 10, 50, and 100 μM, with an increase of 25% observed at 100 μM compared with untreated controls (Figure 5E). A similar trend was observed for total carotenoids in stressed plants, where serotonin application at 10, 50, and 100 μM increased their content relative to untreated stressed plants (Figure S3A). However, no effects of serotonin treatment were observed on shoot biomass or on other physiological parameters, including Fv/Fm and photosynthetic pigment ratios (Figure 5A,D and Figure S3B,C).

In addition, exogenous serotonin at the different concentrations did not affect ABA content under either irrigation regime (Figure 5F). No changes were observed in the other analysed phytohormones (Figure S3E–J) with the exception of a slight increase in GA_4_ in plants treated with 100 μM (Figure S3K) and an increase in SA at 1, 10, and 50 μM (Figure S3D), independently of irrigation regime.

### 3.3 Effects of Serotonin Application Under Water Deficit Conditions in Crop Plants

**FIGURE 6:**
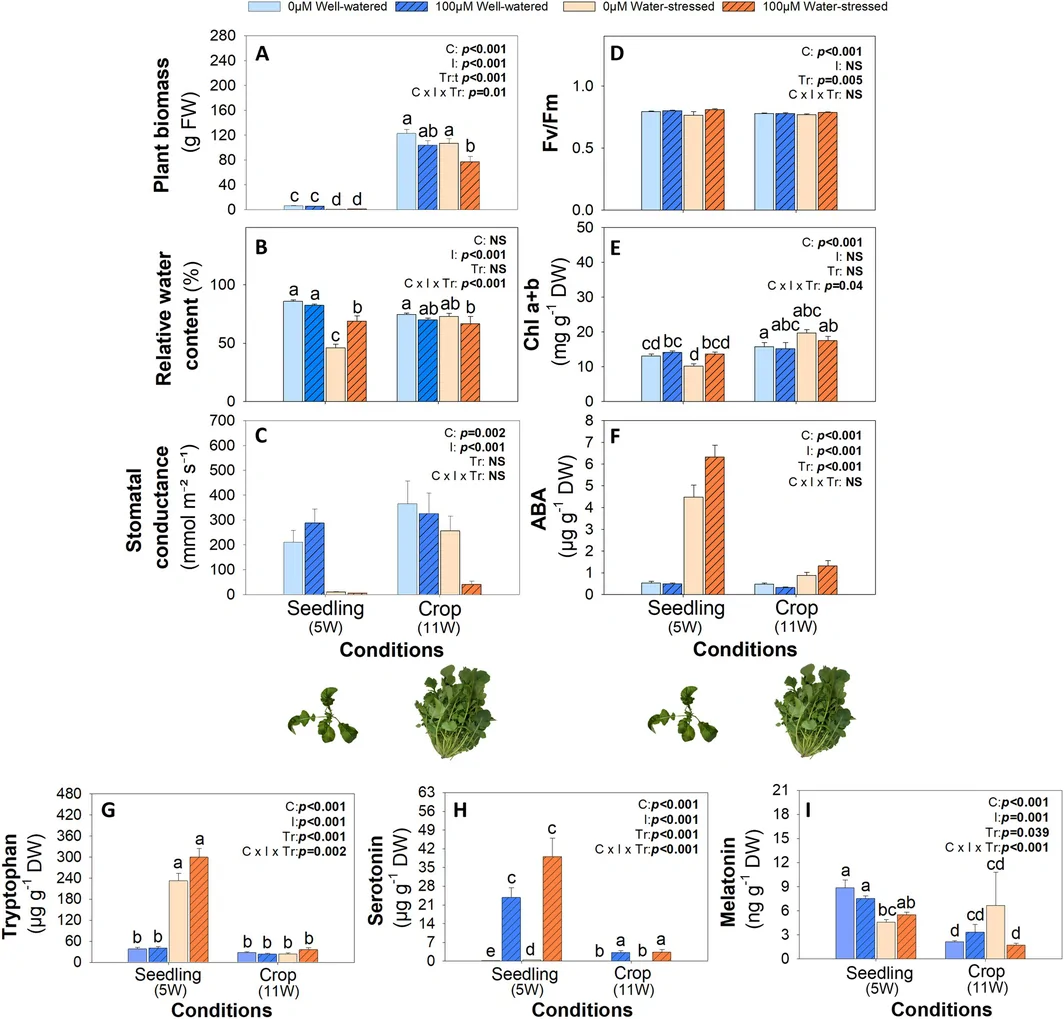
(A) Plant biomass. (B) Relative water content. (C) Stomatal conductance. (D) Maximum efficiency of PSII (Fv/Fm). (E) Total chlorophyll content (Chl a + b). (F) Abscisic acid (ABA) content. (G) Tryptophan content. (H) Serotonin content. (I) Melatonin content in well‐watered and water‐stressed rocket seedlings under semi‐controlled conditions and rocket plants under crop conditions at 14 days and 21 days of water restriction, respectively, and 7 days after serotonin treatment (0, 100 μM). Data are presented as mean ± SE (*n* = 10). Different lowercase letters indicate significant differences (*p* < 0.05, Duncan's post hoc test). C, Condition; I, irrigation; Tr, serotonin treatment; NS indicates no significant differences (*p* > 0.05). Statistical details (*p* values for all main effects and interactions) are provided in Table S1.

Based on the results obtained in seedlings, where 100 μM serotonin showed the most effective response under water stress conditions, this concentration was selected for application in rocket plants grown under field conditions to evaluate its biostimulant effect. However, the results showed that, in contrast to seedlings, where 100 μM serotonin improved relative water content, this effect was not observed under field conditions (Figure 6B). No changes were detected in Fv/Fm or total chlorophyll content (Figure 6D,E). Moreover, serotonin application under water deficit led to a 37% reduction in shoot biomass compared with untreated plants (Figure 6A). In the case of stomatal conductance, significant pairwise interactions were detected between growth condition and serotonin treatment (C × Tr, *p* = 0.011) and between irrigation regime and treatment (I × Tr, *p* = 0.037) (Table S1), but not in the triple interaction (C × I × Tr) (Figure 6C).

Moreover, as observed in seedlings, serotonin application under crop conditions increased endogenous serotonin levels under both irrigation regimes, whereas no changes were detected in tryptophan or melatonin following treatment (Figure 6G–I). Regarding phytohormone levels, ABA showed significant two‐way interactions between growth condition and treatment (C × Tr, *p* = 0.005) and between irrigation and treatment (I × Tr, *p* = 0.022) (Table S1), indicating that serotonin effects on ABA depended on both water availability and growth condition. However, no significant triple interaction (C × I × Tr) was observed (Figure 6F). In the case of other phytohormones, no clear overall effects of serotonin treatment were observed under crop conditions (Figure S4D–K), except for a slight decrease in the cytokinin 2iP in water‐stressed plants treated with serotonin compared with untreated stressed plants (Figure S4I).

**FIGURE 7:**
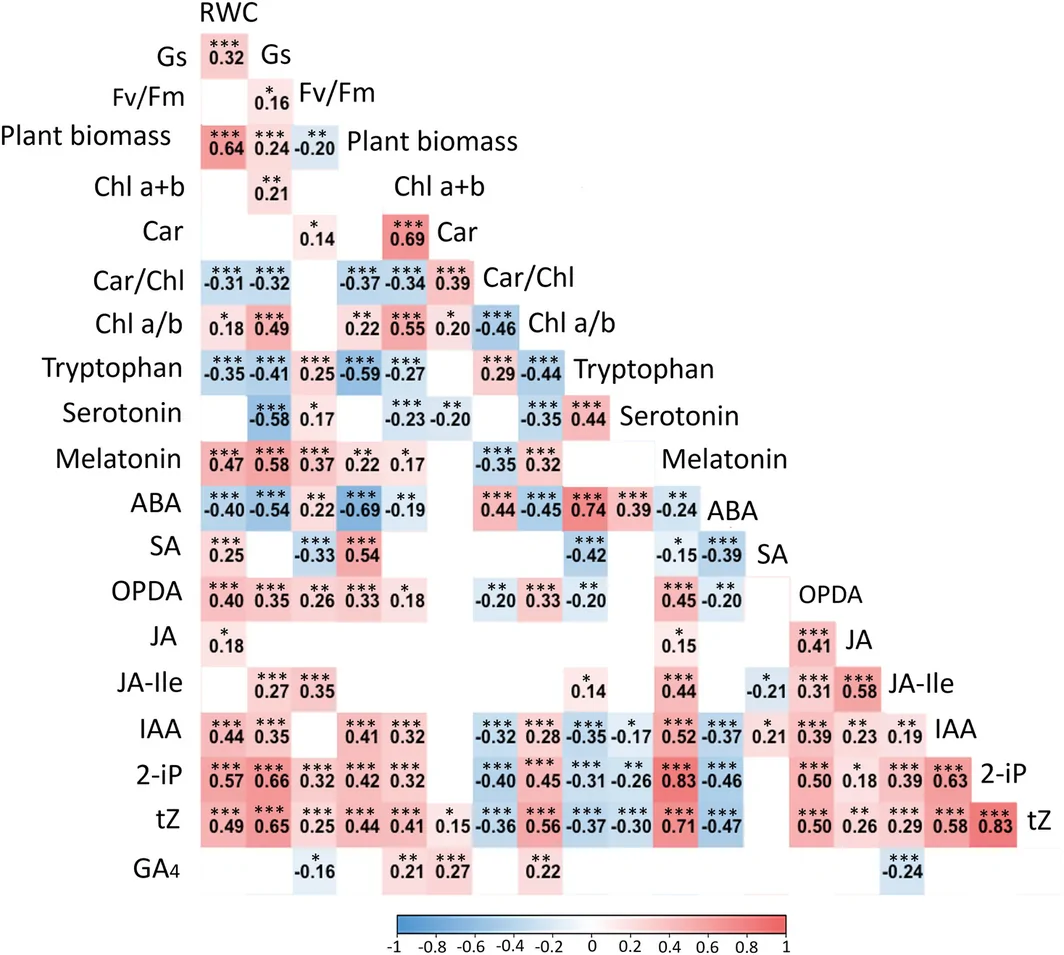
Spearman correlation matrix of physiological parameters, indoleamines and phytohormones concentrations from the three experimental approaches (*n* = 200). The color scale, blue to red, indicates negative and positive correlation coefficients, respectively. Significant correlations are indicated with asterisks, *p < 0.05, **p < 0.01 and ***p < 0.001. No significant correlations are represented as blank space. 2iP, 2‐isopentenyl adenine; ABA, abscisic acid; Car, carotenoids; Chl, chlorophyll; Fv/Fm, maximum efficiency of PSII; GA_4_, Gibberellin 4; gs, stomatal conductance; IAA, indole‐3‐acetic acid; JA, jasmonic acid; JA‐Ile, jasmonoyl isoleucine; OPDA, 12‐*oxo*‐phytodienoic acid; RWC, relative water content; SA, salicylic acid; t‐Z, trans‐zeatin.

Finally, a Spearman correlation matrix including physiological parameters, indoleamines, and phytohormone concentrations from all three experimental approaches (*n* = 200) was performed to further assess relationships among them (Figure 7). When all datasets were combined, serotonin maintained a significant but lower negative correlation with stomatal conductance (*r* = −0.58, *p* < 0.001), although no significant correlation with biomass was observed. In addition, its positive correlation with ABA remained significant (*r* = 0.39, *p* < 0.001), although weaker than that observed in the first experimental approach (*r* = 0.61; Figure S2). In contrast, tryptophan consistently showed correlations with physiological parameters, including stomatal conductance (*r* = −0.41, *p* < 0.001), biomass (*r* = −0.59, *p* < 0.001) and relative water content (*r* = −0.35, *p* < 0.001). It also exhibited a stronger positive correlation with ABA (*r* = 0.74, *p* < 0.001), compared with serotonin and the correlations observed in the first experimental approach (Figures S2 and S7). Moreover, both serotonin and tryptophan showed negative correlations with growth‐related hormones such as IAA and cytokinins, particularly tryptophan, whereas melatonin displayed stronger positive correlations with these hormones (Figure 7).

## 4 Discussion

### 4.1 Drought Stress Impairs Plant Growth and Alters Indoleamine Levels in Rocket Seedlings

Drought is a key abiotic stress that constrains plant growth and productivity by disrupting water relations, photosynthesis, and nutrient uptake, ultimately reducing metabolic efficiency and yield (Zhang et al. 2020; Yi et al. 2016). Adaptation to recurrent drought is a common feature of Mediterranean plant species, which have evolved diverse acclimation strategies to withstand severe water limitation (Bongers et al. 2017). In this study, we provide physiological evidence of acclimation responses to severe drought in rocket plants, with a focus on the involvement of endogenous indoleamines that have been less studied in these processes, such as the case of serotonin. The findings of this study showed that rocket seedlings subjected to 14 days without irrigation exhibited a clear growth‐stress trade‐off, with a marked reduction in shoot biomass associated with decreased gs. This response is consistent with findings in wild rocket ( *Diplotaxis tenuifolia* ), where reduced stomatal conductance has been identified as an early response to stress (Candido et al. 2022). In parallel, drought‐stressed seedlings showed a concomitant decline in leaf water status at peak stress, as reflected by reduced relative water content, a key indicator of plant water status. Similar decreases in relative water content have been reported across 64 rocket genotypes under drought conditions (Nikzad et al. 2023). Indeed, drought responses in rocket were also reflected in a decrease in total chlorophyll content at the stress peak. Photosynthetic pigments are essential for maintaining photosynthetic capacity and thereby influencing plant biomass accumulation. However, stable Fv/Fm values suggest that no significant damage occurred in photosystem II. This may be related to the maintenance of higher total carotenoid levels during the early stages of stress, as well as an increased carotenoid/chlorophyll ratio at the end, which contributes to the protection of the photosynthetic apparatus. Therefore, in rocket seedlings, acclimation to water stress is primarily characterised by reduced biomass due to decreased stomatal conductance and water status, which are partly mediated by a marked increase in ABA levels. ABA is widely recognised as a central regulator of stomatal movements under drought stress, and its signal transduction pathway has been extensively characterised, coordinating stomatal closure to limit water loss and maintain cellular water balance (Ali et al. 2020; Hsu et al. 2021; Yang et al. 2024). Altogether, these responses are consistent with well‐established drought acclimation mechanisms reported across other Mediterranean plant species (Rico et al. 2024; Trovato et al. 2023; Giorio et al. 2018; Rossdeutsch et al. 2016).

However, there is scarce information about the role of tryptophan‐derived indoleamines, particularly serotonin, in plant acclimation to water deficit and even less in rocket plants. Here, our results show that serotonin levels significantly increased in rocket seedlings throughout the drought period compared with irrigated plants. To date, only a limited number of studies have quantified serotonin levels during plant stress responses. Nevertheless, serotonin accumulation has been reported under cold stress in *Kandelia obovata* (Li et al. 2024) and in sunflower seedlings under salt stress (Mukherjee et al. 2014). In contrast, no significant changes in serotonin levels were detected in maize under drought stress (Sun et al. 2020), although this study was based on a single time point. Hence, to our knowledge, this study is the first to report serotonin dynamics under drought stress in rocket. Moreover, tryptophan, the precursor of serotonin, also showed a similar trend, increasing in non‐irrigated plants, with a marked increase at the stress peak. Tryptophan metabolism in plants gives rise to a wide range of bioactive compounds, including auxin, serotonin, melatonin, but also indole glucosinolates, and phytoalexins, which are known to play key roles in plant growth regulation and defence responses under stress conditions (Wang et al. 2025). In this context, a previous study demonstrated that exogenous tryptophan enhanced cold stress tolerance in strawberry flowers, increasing serotonin and N‐acetylserotonin, indicating a rapid metabolic conversion of tryptophan into downstream metabolites in response to stress (Ayyanath et al. 2024). On the other hand, melatonin levels remained unchanged throughout the drought period. It has been shown that environmental stress can increase endogenous melatonin levels in plants, such as in sunflower seedlings under salt stress or in alfalfa plants under waterlogging stress (Mukherjee et al. 2014; Zhang et al. 2019). However, other studies did not observe changes in melatonin content under drought conditions (Fleta‐Soriano et al. 2017). In this context, Yolcu et al. (2025) demonstrated that expression patterns of melatonin biosynthetic genes vary depending on tissue type, genotype, and the type of abiotic stress, which may be one reason why melatonin levels do not always change, although its role in abiotic stress tolerance has been widely described (Ahmad et al. 2023; Tiwari et al. 2021).

Therefore, rocket plants subjected to severe water deficit appear to exhibit an acclimation response characterised by a strong reduction in growth and gs, followed by a decrease in relative water content. This response is accompanied by marked increases in ABA, as well as in serotonin and its precursor tryptophan, but not melatonin. Moreover, correlation analyses revealed consistent negative associations between serotonin and tryptophan with stress‐related parameters, particularly stomatal conductance and shoot biomass. ABA also showed a positive correlation with serotonin, probably reflecting the concomitant accumulation of both metabolites during drought progression. This is consistent with the well‐established increase in ABA biosynthesis under drought stress, which activates adaptive responses to maintain plant water status, together with the increase in endogenous serotonin observed in the present study, which may also represent part of the plant stress response. Interestingly, tryptophan followed a similar accumulation pattern but showed an even stronger correlation with ABA, suggesting a closer association between tryptophan metabolism and drought responses in rocket plants.

### 4.2 Dose‐Dependent Serotonin Effects on Drought Tolerance Improve Water Status but Do Not Alter ABA Accumulation

To further investigate the involvement of serotonin in plant responses to water deficit, rocket seedlings were treated with different concentrations of serotonin (1, 10, 50, and 100 μM) to evaluate its effects. As expected, endogenous serotonin levels increased in a dose‐dependent manner, reaching a maximum at 100 μM in both irrigated and stressed plants. Such increases following exogenous application have been previously reported (Arabia et al. 2026; Pelagio‐Flores et al. 2011). Surprisingly, exogenous serotonin did not lead to changes in melatonin content, nor did it affect tryptophan levels. These findings suggest that, although serotonin accumulates markedly, this increase is not coupled to enhanced melatonin biosynthesis, indicating a possible limitation in its downstream conversion, while tryptophan levels remain unaffected, with no apparent feedback on upstream metabolism.

The application of serotonin at 100 μM led to the most pronounced and effective results to enhance water stress tolerance, increasing the relative water content in stressed plants compared with untreated plants. Moreover, the maintenance of plant water status was associated with stomatal closure, as reflected by the lowest stomatal conductance values observed in stressed rocket plants treated with the highest serotonin dose (100 μM). These results are consistent with a study in saffron, which showed that foliar application of serotonin at 100 μM improved the relative water content under drought conditions (Pontes et al. 2024). Moreover, Akcay and Okudan (2023) demonstrated the protective effect of exogenous serotonin at 5 μM against drought, maintaining the relative water content at levels comparable to control plants. In a recent review by Sun et al. (2025), the effects of exogenous serotonin in plants under stress conditions were summarised, reporting a wide range of applied concentrations, from low levels such as 1 μM to higher doses up to 300 μM, depending on species and stress type. For example, Zhao et al. (2023) tested several doses (1–3.0 mg L^−1^) of serotonin in maize seedlings under deep‐seeding stress and found that 2.5 mg L^−1^ (~14.2 μM) was the most effective dose for improving stress tolerance, which is approximately seven times lower than the optimal dose in the present study. In contrast, Liu et al. (2021) applied 50, 100, 200, and 300 μM serotonin via nutrient solution to assess its effects under salt stress in rapeseed and reported that 200 μM provided the highest level of protection against salinity. A similar dose‐dependent trend, but with a negative outcome at higher concentrations, was reported by Tian et al. (2019), who observed that when the application dose exceeds 150 μM in rapeseed under drought stress, biomass is negatively affected. Similar results were also observed in root development, where serotonin at lower concentrations (10–160 μM) stimulated lateral root growth, whereas higher doses restricted it (Pelagio‐Flores et al. 2011). Hence, the effects of optimal and suboptimal serotonin concentrations on plant growth and stress tolerance vary considerably among stress types, species and organs, highlighting the importance of dose–response relationships and warranting further investigation (Sun et al. 2025).

Furthermore, given the central role of ABA in regulating stomatal closure and maintaining leaf water status during drought, we investigated whether the beneficial effects of exogenous serotonin were associated with changes in endogenous ABA accumulation. However, no changes in ABA levels were detected between serotonin‐treated and control plants under drought conditions, nor was any increase in shoot biomass observed. This lack of variation in ABA content suggests that the beneficial effects of exogenous serotonin are unlikely to be mediated through increased ABA accumulation. This interpretation is consistent with the findings of Shukla et al. (2021), who reported that exogenous serotonin improved salt stress tolerance in Arabidopsis without inducing the ABA‐responsive transcription factors ABI3 and ABI5, which are typically induced under drought stress. In contrast, melatonin promoted the expression of these ABA‐responsive genes under the same experimental conditions, suggesting that the two indoleamines may promote stress tolerance through different mechanisms. Therefore, our data further support the hypothesis that serotonin may, at least in part, act through ABA‐independent pathways. However, further studies addressing additional components of the ABA signaling pathway will be necessary to confirm this interpretation. Potential mechanisms underlying these responses may include the activation of antioxidant defence systems, as reported in previous studies (He et al. 2021; Akcay and Okudan 2023; Gavyar et al. 2024; Pontes et al. 2024), as well as the accumulation of non‐enzymatic antioxidant compounds such as carotenoids. In the present study, serotonin increased the content of photosynthetic pigments, including carotenoids, in a dose‐dependent manner under severe drought. These compounds enhance photoprotection through their antioxidant activity and by dissipating excess excitation energy in photosystem II, which helps protect the photosynthetic apparatus and reduces ROS accumulation (Young 1991; Demmig‐Adams and Adams 1996). This may help maintain membrane integrity and, indirectly, plant water status under stress conditions. Consistently, previous studies have also reported that exogenous serotonin increases carotenoid content under salinity and drought stress at 200 and 100 μM, respectively (Zahra et al. 2024; Gavyar et al. 2024). However, the specific mechanisms by which serotonin modulates photosynthetic pigments remain unclear and have often been attributed to pathways shared with melatonin, given that serotonin is a metabolic intermediate in its biosynthetic route. Therefore, further research is required to elucidate the molecular mechanisms underlying these effects.

### 4.3 Limited Effects of Serotonin Application on Drought Responses Under Field Conditions

Given the beneficial effects of exogenous serotonin at 100 μM in rocket seedlings, particularly in improving the relative water content under drought conditions, it is relevant to consider whether such responses could also occur under field conditions. In this context, serotonin may have potential as a biostimulant in crops exposed to limited water availability, contributing to improved plant water status and potentially reducing irrigation requirements. For this reason, serotonin application at 100 μM was evaluated under field conditions in rocket plants under both well‐watered and reduced water availability regimes. Contrary to expectations, serotonin application under crop (field) conditions did not enhance relative water content compared with the effects observed in seedlings. These differences may be related to the phenological stage of the plants at the time of application, as physiological responsiveness to biostimulants can vary across developmental stages. In addition, although the reduced irrigation treatment induced a physiological response to water deficit, as evidenced by the significant changes in chlorophyll content and increased ABA levels in water‐stressed plants compared with well‐watered plants under field conditions (Table S2), the water stress perceived by the plants was less severe than in the controlled seedling experiment, as reflected by the absence of significant differences in biomass and relative water content. Despite 21 days without irrigation before harvest, the lower stress severity was likely due to the greater water‐holding capacity under field conditions and the more advanced phenological stage of the plants. Therefore, the lower severity of water deficit under field conditions may explain why the beneficial effects of serotonin observed in seedlings were not reproduced under field conditions and may even have contributed to the slight reduction in biomass. Indeed, previous studies have reported limited or even negative effects of serotonin under certain conditions, such as the negligible impact on yield described by Akcay and Okudan (2023) in tomato plants under salinity stress, or the detrimental effects observed at high doses in rapeseed under cold stress (He et al. 2021). Moreover, under crop conditions, endogenous serotonin levels did not increase under reduced water availability compared with irrigated plants, in contrast to the response observed in seedlings, where serotonin increased under drought. This supports the idea that serotonin involvement may be more pronounced at early developmental stages and/or under more severe stress conditions. Overall, these findings indicate that the effects of exogenous serotonin on drought tolerance depend strongly on application dose, growth conditions, and the phenological stage of the plant. Therefore, validation under field conditions is essential, as responses may differ substantially from those observed under controlled environments.

### 4.4 Tryptophan Exhibits a Stronger Association With ABA Accumulation Than Serotonin Under Water Deficit Conditions in Rocket Plants

To further assess the relationships between indoleamines, physiological stress markers, and phytohormones, a Spearman correlation matrix was generated using all available data (*n* = 200) from the three experimental approaches (both endogenous and exogenous). Overall, when all datasets were integrated, the strength of the associations involving serotonin decreased, particularly with stomatal conductance and ABA, suggesting a less consistent relationship with physiological stress responses across conditions, especially in relation to ABA. In contrast, tryptophan showed more consistent negative correlations with physiological parameters and a stronger positive correlation with ABA content (*r* = 0.74), compared with serotonin (*r* = 0.39), suggesting a more robust and stable association with ABA accumulation across experimental approaches.

Although endogenous serotonin and ABA accumulated concomitantly during drought, the association between both metabolites became considerably weaker when endogenous measurements were analysed together with the exogenous serotonin experiments. Furthermore, exogenous serotonin application did not alter endogenous ABA levels under any of the conditions evaluated. Together, these findings do not support a close functional relationship between serotonin and ABA in rocket plants. Current evidence regarding serotonin‐ABA interactions remains limited and appears to be highly context‐dependent across species and biological processes. For example, Lu et al. (2022) identified that ABA represses the expression of the serotonin biosynthetic gene *OsT5H*, thereby promoting root suberisation as an adaptive response to salinity stress in rice and Arabidopsis. Conversely, increased serotonin biosynthesis attenuated suberin deposition and reduced salt tolerance, indicating that serotonin acts downstream of ABA as a negative regulator of this ABA‐dependent adaptive mechanism. More recently, Cui et al. (2025) reported a bidirectional ABA‐serotonin regulatory loop in rice, in which ABA induces serotonin biosynthesis via ABI5‐dependent gene activation, while serotonin feeds back to attenuate ABA signaling, leading to reduced ABI5 phosphorylation, and thus limiting ABA‐induced serotonin biosynthesis. On the other hand, as discussed above, Shukla et al. (2021) reported that exogenous serotonin enhanced salt stress tolerance in Arabidopsis without inducing the expression of ABA‐responsive genes, including ABI3 and ABI5. Overall, the available evidence suggests that serotonin may contribute to abiotic stress tolerance through both ABA‐dependent and ABA‐independent mechanisms depending on the biological context, highlighting the need for further mechanistic studies. Consistent with this complexity, our results in *Eruca vesicaria* subsp. *sativa* do not support a major role for serotonin in regulating ABA accumulation under drought conditions.

Likewise, although serotonin and IAA share tryptophan as a common precursor and previous studies have suggested potential interactions between them, particularly in roots (Pelagio‐Flores et al. 2011), our results do not support such a relationship in rocket, as exogenous serotonin application did not alter IAA content under any of the tested conditions and only a weak correlation was observed. These findings, together with the lack of evidence for a specific serotonin receptor or dedicated signal transduction pathway in plants, suggest that serotonin may contribute to stress responses through mechanisms that remain to be fully elucidated. Hence, in rocket plants under water deficit conditions, its role appears to be more closely associated with metabolic regulation and indoleamine homeostasis rather than with direct modulation of ABA or IAA levels.

Instead, our results indicate that tryptophan shows a stronger association with ABA than serotonin under water deficit conditions. However, whether this reflects a direct regulatory role of tryptophan or a coordinated response of both metabolites to upstream drought signals cannot be determined from the present study and requires further investigation. In Arabidopsis, Liu et al. (2022) showed that tryptophan metabolism is closely integrated with ABA signaling through tryptophan synthase β subunit 1 (TSB1), which coordinates the balance between tryptophan availability and ABA homeostasis, linking growth regulation with abiotic stress responses. However, this relationship has not yet been explored in rocket under drought conditions. Moreover, tryptophan also showed a significant negative correlation with IAA content, suggesting that the relationship between tryptophan metabolism and auxin homeostasis may be relevant under water deficit conditions. Given that tryptophan is also a precursor of IAA, changes in tryptophan metabolism under drought conditions could influence auxin biosynthesis and, consequently, growth‐related processes. Further studies are therefore needed to clarify the involvement of tryptophan metabolism in drought stress responses in rocket plants, including its potential contribution to ABA regulation and auxin‐mediated growth responses, which could be relevant for understanding and improving stress resilience in agriculturally important leafy crops.

## 5 Conclusions

In conclusion, our findings indicate that under water deficit conditions, rocket plants exhibit increased endogenous levels of serotonin and its precursor tryptophan. Both compounds were negatively correlated with physiological stress markers and positively correlated with ABA content, which showed a similar pattern of accumulation during drought progression. Furthermore, exogenous application of serotonin improved plant water status in seedlings in a dose‐dependent manner, with 100 μM being the most effective treatment. However, these effects were not accompanied by changes in ABA levels and were not reproduced under field conditions. When all datasets were combined in a correlation matrix, the associations involving serotonin became weaker and tryptophan showed a stronger and more consistent association with ABA and stress‐related parameters. Overall, under the conditions evaluated in this study, our results do not support a central role for serotonin in regulating ABA‐mediated drought responses in rocket plants. Future studies examining additional components of the ABA signaling pathway will be necessary to better understand if serotonin influences ABA‐dependent responses. Complementary approaches, such as serotonin application before drought onset, together with research under different environmental conditions, developmental stages, and in additional plant species, will help determine the broader relevance of these findings and clarify whether serotonin plays a role under drought conditions comparable to that proposed for other tryptophan‐derived signaling molecules, such as melatonin or auxin. Finally, direct manipulation of tryptophan metabolism, including exogenous application of tryptophan and IAA, biosynthesis inhibition, or the use of biosynthetic mutants, will be essential to determine whether tryptophan association with ABA and IAA reflects a direct regulatory role or coordinated responses to drought.

## Author Contributions

S.Á.‐R., S.M.‐B. and A.A. conceptualised and designed the experiments. S.Á.‐R. and A.A. performed the experiments and S.Á.‐R. analysed the data. S.Á.‐R. and A.A. wrote the first draft of the manuscript. S.M.‐B. revised the manuscript. All authors read and approved the final manuscript. S.M.‐B. was responsible for funding acquisition.

## Funding

This work was supported by the grant INTERACT 2024 programme from the Institut de Recerca en Nutrició i Seguretat Alimentària (INSA‐UB), funded under the María de Maeztu Units of Excellence Programme (CEX2021‐001234‐M; MCIN/AEI/10.13039/501100011033).

## Conflicts of Interest

The authors declare no conflicts of interest.

## Supporting information


**Table S1:** ppl71106‐sup‐0001‐Supinfo.pdf. *p* values obtained from a three‐way ANOVA assessing the effects of Condition (C: seedling, crop), Irrigation (I: well‐watered, water‐stressed), and Treatment (Tr: 0 and 100 μM serotonin), as well as their two‐way and three‐way interactions, on physiological and hormonal parameters. NS indicates non‐significant effects (*p* > 0.05). RWC, relative water content; gs, stomatal conductance; Chl, chlorophyll; Car, carotenoids; Trp, tryptophan; Ser, serotonin; Mel, melatonin; ABA, abscisic acid; SA, salicylic acid; OPDA, 12‐*oxo*‐phytodienoic acid; JA, jasmonic acid; JA‐Ile, jasmonoyl isoleucine; IAA, indole‐3‐acetic acid; 2iP, 2‐isopentenyl adenine; t‐Z, trans‐zeatin; GA4, Gibberellin 4.
**Table S2:** Comparison of physiological stress markers between well‐watered and water stressed rocket plants under field conditions at harvest. *p* values indicate the significance of the comparison between irrigation treatments. NS indicates no significant differences (*p* > 0.05).
**Figure S1:** (A) Total carotenoid content (Car). (B) Carotenoid/chlorophyll ratio (Car/Chl). (C) Chlorophyll a/b ratio (Chl a/b). (D) 12‐oxo‐phytodienoic acid (OPDA). (E) Jasmonic acid (JA). (F) Jasmonoyl‐isoleucine (JA‐Ile). (G) 2‐Isopentenyl adenine (2iP). (H) Trans zeatin (t‐Z). (I) Gibberellin 4 (GA4) content in well‐watered and water‐stressed rocket plants after 7, 10 and 14 days of water restriction. Data are presented as mean ± SE (*n* = 10). Different lowercase letters indicate significant differences when the Time (T) × Irrigation (I) interaction was significant (*p* < 0.05, Duncan's post hoc test), and different uppercase letters indicate significant differences among Time levels (*p* < 0.05, Duncan's post hoc test). NS indicates no significant differences (*p* > 0.05).
**Figure S2:** Spearman correlation matrix of physiological parameters, indoleamines and phytohormones concentrations after 7, 10 and 14 days of water restriction in well‐watered and water‐stressed plants (*n* = 60). The colour scale, blue to red, indicates negative and positive correlation coefficients, respectively. Significant correlations are indicated with asterisks, *p < 0.05, **p < 0.01, and ***p < 0.001. No significant correlations are represented as blank space. RWC, relative water content; gs, stomatal conductance; Fv/Fm, maximum efficiency of PSII; Chl, chlorophyll; Car, carotenoids; ABA, abscisic acid; SA, salicylic acid; OPDA, 12‐oxo‐phytodienoic acid; JA, jasmonic acid; JA‐Ile, jasmonoyl isoleucine; IAA, indole‐3‐acetic acid; 2iP, 2‐isopentenyl adenine; t‐Z, trans zeatin; GA4, Gibberellin 4.
**Figure S3:** (A) Total carotenoid content (Car). (B) Carotenoid/chlorophyll ratio (Car/Chl). (C) Chlorophyll a/b ratio (Chl a/b). (D) Salicylic acid (SA). (E) 12‐oxo‐phytodienoic acid (OPDA). (F) Jasmonic acid (JA). (G) Jasmonoyl‐isoleucine (JA‐Ile). (H) Indole‐3‐acetic acid (IAA). (I) 2‐Isopentenyl adenine (2iP). (J) Trans‐zeatin (t‐Z). (K) Gibberellin 4 (GA4) content after 14 days of water restriction and 7 days after serotonin treatment (0, 1, 10, 50, and 100 μM) in well‐watered and water‐stressed plants. Data are presented as mean ± SE (*n* = 10). Different lowercase letters indicate significant differences (p < 0.05, Duncan's post hoc test). I, irrigation; Tr, serotonin treatment; NS indicates no significant differences (p > 0.05). **Figure S4:** (A) Total carotenoid content (Car). (B) Carotenoid/chlorophyll ratio (Car/Chl). (C) Chlorophyll a/b ratio (Chl a/b). (D) Salicylic acid (SA). (E) 12‐oxo‐phytodienoic acid (OPDA). (F) Jasmonic acid (JA). (G) Jasmonoyl‐isoleucine (JA‐Ile). (H) Indole‐3‐acetic acid (IAA). (I) 2‐Isopentenyl adenine (2iP). (J) Trans‐zeatin (t‐Z). (K) Gibberellin 4 (GA4) content in well‐watered and water‐stressed rocket seedlings under semi‐controlled conditions and rocket plants under crop conditions after 14 and 21 days of water restriction, respectively, and 7 days after serotonin treatment (0 and 100 µM). Data are presented as mean ± SE (*n* = 10). Different lowercase letters indicate significant differences (*p* < 0.05, Duncan’s post‐hoc test). C, Condition; I, irrigation; Tr, serotonin treatment; NS indicates no significant differences (*p* > 0.05). Statistical details (*p*‐values for all main effects and interactions) are provided in Table S1.

## Data Availability

The data that support the findings of this study are available on request from the corresponding author.
