# Supplementary material for: Dissecting the Role of Serotonin in Drought Responses of Rocket Plants: From Serotonin Accumulation to Tryptophan–ABA Associations
Source: Physiol Plant. 2026 Sep 4;178(5):e71106. doi: 10.1111/ppl.71106 (PMC13545333; doi:10.1111/ppl.71106)
Supplement: Supplementary file 1 [file PPL-178-e71106-s001.pdf]

Dissecting the role of serotonin in drought responses of rocket plants: from serotonin accumulation to tryptophan–ABA associations

Sofía Álvarez-Real, Sergi Munné-Bosch, Alba Arabia

Supplementary Tables

Table S1. *P*-values obtained from a three-way ANOVA assessing the effects of Condition (C: seedling, crop), Irrigation (I: well-watered, water-stressed), and Treatment (Tr: 0 and 100 µM serotonin), as well as their two-way and three-way interactions, on physiological and hormonal parameters. NS indicates non-significant effects ($p > 0.05$). RWC, relative water content; gs, stomatal conductance; Chl, chlorophyll; Car, carotenoids; Trp, tryptophan; Ser, serotonin; Mel, melatonin; ABA, abscisic acid; SA, salicylic acid; OPDA, 12-*oxo*-phytodienoic acid; JA, jasmonic acid; JA-Ile, jasmonoyl isoleucine; IAA, indole-3-acetic acid; 2iP, 2-isopentenyl adenine; t-Z, trans-zeatin; GA_4, Gibberellin 4.

**Parameters**	**Experimental factors**						
	C	I	Tr	C x I	C x Tr	I x Tr	C x I x Tr
Plant biomass	*p*<0.001	*p*<0.001	*p*<0.001	*p*=0.005	*p*=0.012	*p*<0.001	*p*=0.010
RWC	NS	*p*<0.001	NS	*p*<0.001	*p*<0.001	*p*<0.001	*p*<0.001
gs	*p*=0.002	*p*<0.001	NS	NS	*p*=0.011	*p*=0.037	NS
Fv/Fm	*p*<0.001	NS	*p*=0.005	NS	*p*=0.024	NS	NS
Chl a+b	*p*<0.001	NS	NS	*p*<0.001	*p*=0.016	NS	p=0.039
Car	*p*<0.001	*p*<0.001	NS	NS	NS	*p*<0.001	NS
Car/Chl	NS	*p*<0.001	NS	NS	NS	*p*<0.001	*p*=0.024
Chl a/b	*p*<0.001	*p*=0.011	*p*<0.001	*p*<0.001	*p*<0.001	*p*<0.001	*p*<0.001
Trp	*p*<0.001	*p*<0.001	*p*<0.001	*p*<0.001	*p*<0.001	*p*<0.001	*p*=0.002
Ser	*p*<0.001	*p*<0.001	*p*<0.001	*p*<0.001	*p*<0.001	*p*<0.001	*p*<0.001
Mel	*p*<0.001	*p*=0.001	*p*=0.039	*p*<0.001	*p*=0.016	NS	*p*<0.001
ABA	*p*<0.001	*p*<0.001	*p*<0.001	NS	*p*=0.005	*p*=0.022	NS
SA	*p*<0.001	*p*=0.004	NS	*p*=0.005	*p*=0.031	NS	NS
OPDA	NS	*p*=0.001	*p*<0.001	*p*<0.001	NS	NS	*p*<0.001
JA	*p*=0.012	NS	*p*=0.013	*p*=0.001	*p*=0.024	NS	NS
JA-Ile	*p*<0.001	NS	NS	NS	NS	NS	NS
IAA	*p*<0.001	NS	NS	*p*<0.001	NS	NS	NS
GA_4	*p*<0.001	NS	*p=0.005*	NS	NS	NS	NS
2-iP	*p*<0.001	*p*<0.001	*p*=0.016	*p*<0.001	*p*=0.022	NS	*p*=0.012
tZ	*NS*	*p*=0.038	*p*<0.001	*p*<0.001	*p*<0.001	*p*<0.001	*p*=0.002

Table S2. Comparison of physiological stress markers between well-watered and water-stressed rocket plants under field conditions at harvest. *p*-values indicate the significance of the comparison between irrigation treatments. NS indicates no significant differences ($p > 0.05$).

Irrigation treatment	Biomass (g FW)	RWC (%)	gs (mmol m^{-2} s^{-1})	Fv/Fm	Chl a+b (mg g^{-1} DW)	ABA (μg g^{-1} DW)
Well-watered	122.63±6.36	74.67±1.18	364.86±91.36	0.78±0.005	15.79±1.19	0.47±0.07
Water-stressed	106.90±7.46	72.99±2.67	255.87±59.73	0.77±0.006	19.70±0.96	0.87±0.15
p-value	NS	NS	NS	NS	0.020	0.011

Supplementary Figures

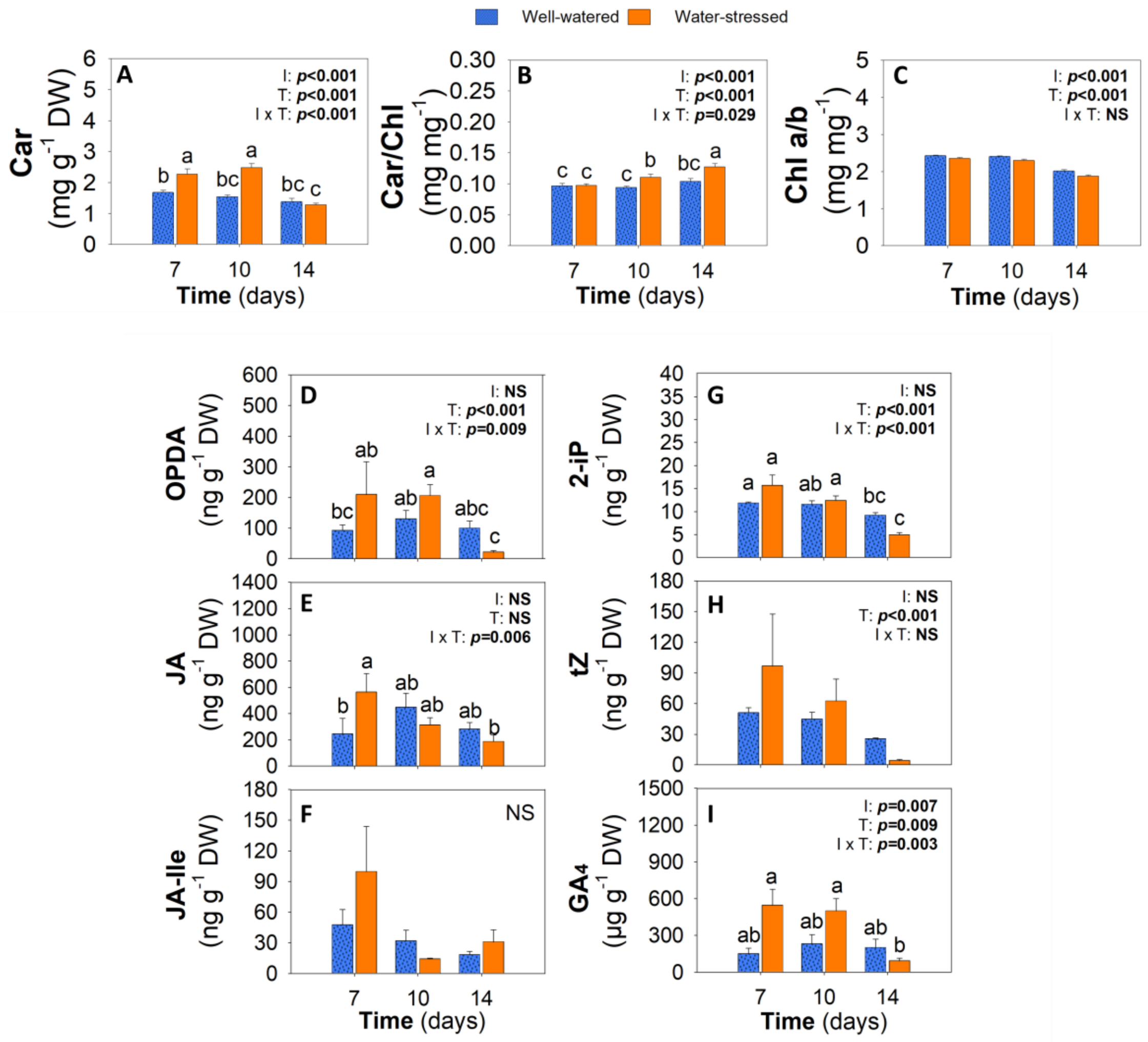

Figure S1. (A) Total carotenoid content (Car). (B) Carotenoid/chlorophyll ratio (Car/Chl). (C) Chlorophyll a/b ratio (Chl a/b). (D) 12-*oxo*-phytodienoic acid (OPDA). (E) Jasmonic acid (JA). (F) Jasmonoyl-isoleucine (JA-Ile). (G) 2-Isopentenyl adenine (2iP). (H) Trans-zeatin (t-Z). (I) Gibberellin 4 (GA_4) content in well-watered and water-stressed rocket plants after 7, 10 and 14 days of water restriction. Data are presented as mean ± SE (n = 10). Different lowercase letters indicate significant differences when the Time (T) × Irrigation (I) interaction was significant ($p < 0.05$, Duncan's post-hoc test), and different uppercase letters indicate significant differences among Time levels ($p < 0.05$, Duncan's post-hoc test). NS indicates no significant differences ($p > 0.05$).

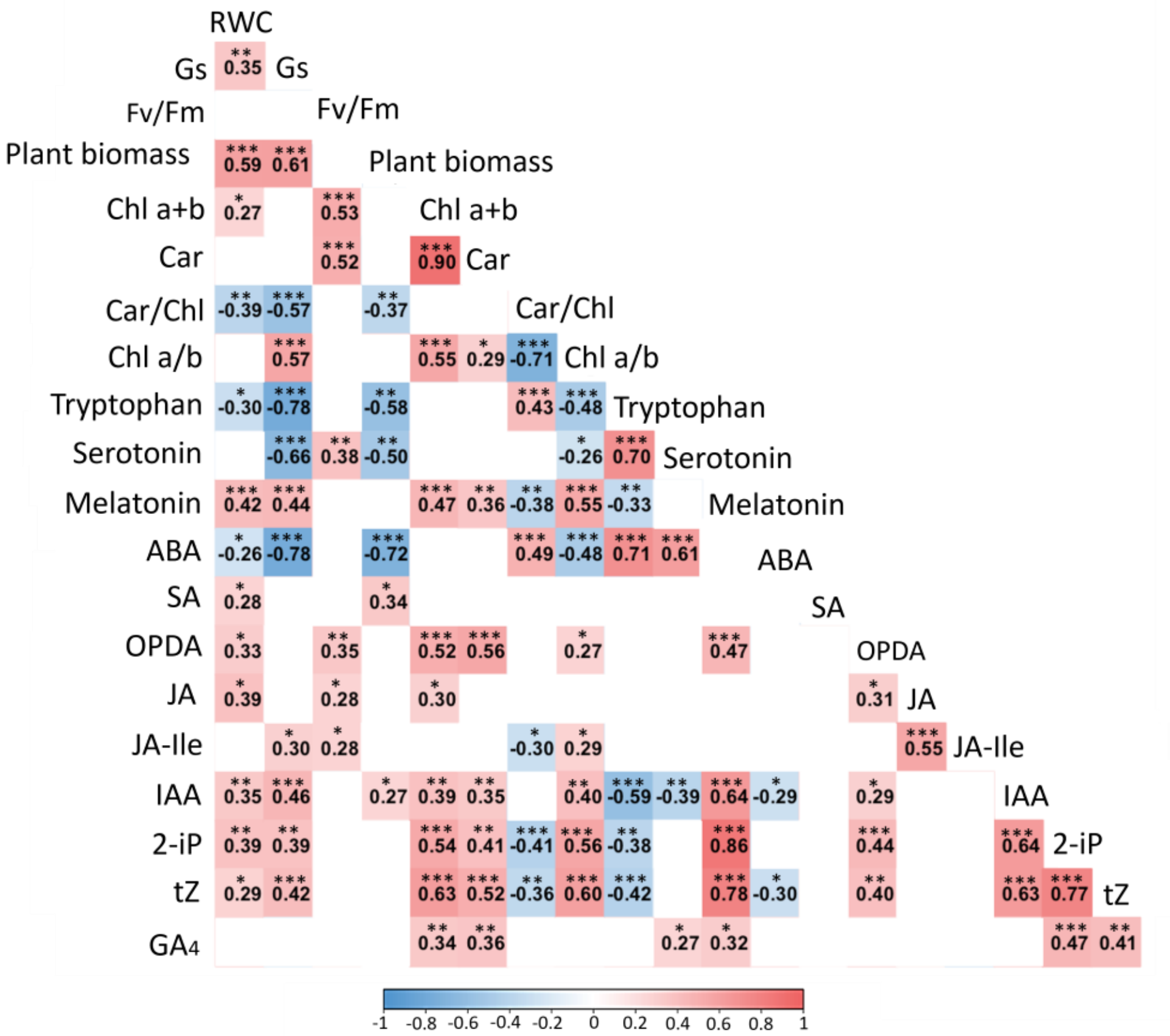

Figure S2. Spearman correlation matrix of physiological parameters, indoleamines and phytohormones concentrations after 7, 10 and 14 days of water restriction in well-watered and water-stressed plants (*n* = 60). The colour scale, blue to red, indicates negative and positive correlation coefficients, respectively. Significant correlations are indicated with asterisks, *($p < 0.05$), ** ($p < 0.01$) and ***($p < 0.001$). No significant correlations are represented as blank space. RWC, relative water content; gs, stomatal conductance; Fv/Fm, maximum efficiency of PSII; Chl, chlorophyll; Car, carotenoids; ABA, abscisic acid; SA, salicylic acid; OPDA, 12-*oxo*-phytodienoic acid; JA, jasmonic acid; JA-Ile, jasmonoyl isoleucine; IAA, indole-3-acetic acid; 2iP, 2-isopentenyl adenine; t-Z, trans-zeatin; GA_4, Gibberellin 4.

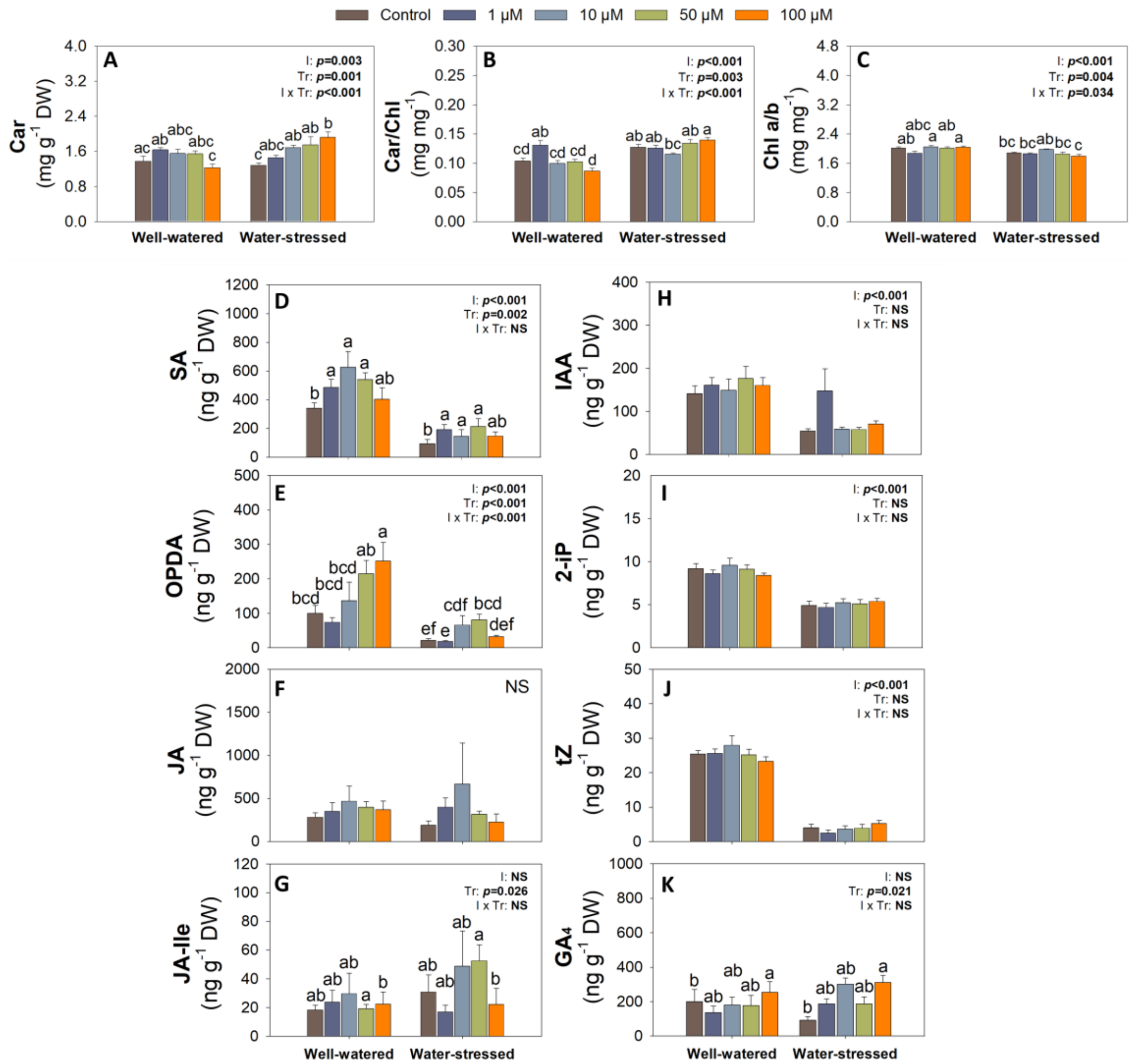

Figure S3. (A) Total carotenoid content (Car). (B) Carotenoid/chlorophyll ratio (Car/Chl). (C) Chlorophyll a/b ratio (Chl a/b). (D) Salicylic acid (SA). (E) 12-*oxo*-phytodienoic acid (OPDA). (F) Jasmonic acid (JA). (G) Jasmonoyl-isoleucine (JA-Ile). (H) Indole-3-acetic acid (IAA). (I) 2-Isopentenyl adenine (2iP). (J) Trans-zeatin (t-Z). (K) Gibberellin 4 (GA_4) content after 14 days of water restriction and 7 days after serotonin treatment (0, 1, 10, 50, and 100 µM) in well-watered and water-stressed plants. Data are presented as mean ± SE (n = 10). Different lowercase letters indicate significant differences ($p < 0.05$, Duncan's post-hoc test). I, irrigation; Tr, serotonin treatment; NS indicates no significant differences ($p > 0.05$).

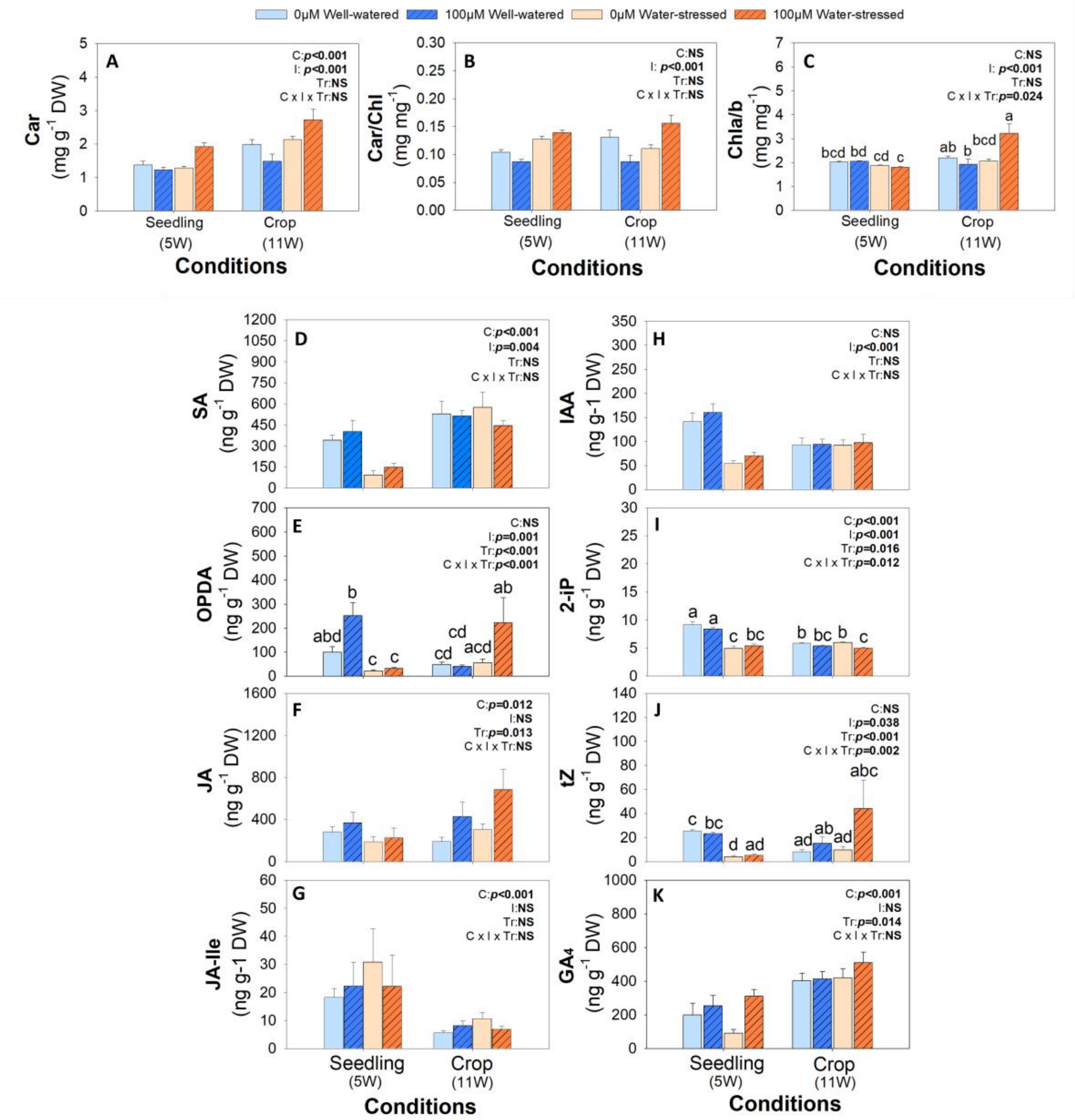

Figure S4. (A) Total carotenoid content (Car). (B) Carotenoid/chlorophyll ratio (Car/Chl). (C) Chlorophyll a/b ratio (Chl a/b). (D) Salicylic acid (SA). (E) 12-*oxo*-phytodienoic acid (OPDA). (F) Jasmonic acid (JA). (G) Jasmonoyl-isoleucine (JA-Ile). (H) Indole-3-acetic acid (IAA). (I) 2-Isopentenyl adenine (2iP). (J) Trans-zeatin (t-Z). (K) Gibberellin 4 (GA_4) content in well-watered and water-stressed rocket seedlings under semi-controlled conditions and rocket plants under crop conditions after 14 and 21 days of water restriction, respectively, and 7 days after serotonin treatment (0 and 100 µM). Data are presented as mean ± SE (n = 10). Different lowercase letters indicate significant differences ($p < 0.05$, Duncan's post-hoc test). C, Condition; I, irrigation; Tr, serotonin treatment; NS indicates no significant differences ($p > 0.05$). Statistical details (p-values for all main effects and interactions) are provided in Table S1.
